# Patient-Specific Blood Flow Analysis for Cerebral Arteriovenous Malformation Based on Digital Subtraction Angiography Images

**DOI:** 10.3389/fbioe.2020.00775

**Published:** 2020-07-23

**Authors:** Changwei Zhang, Nixon Chau, Harvey Ho

**Affiliations:** ^1^Department of Neurosurgery, West China Hospital, Sichuan University, Chengdu, China; ^2^Auckland Bioengineering Institute, The University of Auckland, Auckland, New Zealand

**Keywords:** arterial venous malformation (AVM), digital subtraction angiography (DSA), computer model, cerebral circulation, brain, autoregulation

## Abstract

Real-time digital subtraction angiography (DSA) is capable of revealing the cerebral vascular morphology and blood flow perfusion patterns of arterial venous malformations (AVMs). In this study, we analyze the DSA images of a subject-specific left posterior AVM case and customize a generic electric analog model for cerebral circulation accordingly. The generic model consists of electronic components representing 49 major cerebral arteries and veins, and yields their blood pressure and flow rate profiles. The model was adapted by incorporating the supplying and draining patterns of the AVM to simulate some typical AVM features such as the blood “steal” syndrome, where the flow rate in the left posterior artery increases by almost three times (∼300 ml/min vs 100 ml/min) compared with the healthy case. Meanwhile, the flow rate to the right posterior artery is reduced to ∼30 ml/min from 100 ml/min despite the presence of an autoregulation mechanism in the model. In addition, the blood pressure in the draining veins is increased from 9 to 22 mmHg, and the blood pressure in the feeding arteries is reduced from 85 to 30 mmHg due to the fistula effects of the AVM. In summary, a first DSA-based AVM model has been developed. More subject-specific AVM cases are required to apply the presented *in silico* model, and *in vivo* data are used to validate the simulation results.

## Introduction

In clinical practices, the real-time flow of radiopaque agents revealed from digital subtraction angiography (DSA) images assists clinicians in determining the anatomical features of arteriovenous malformations (AVMs), which are a tangle of vessels (nidus) connecting cerebral arteries and veins, bypassing the capillary bed (Spetzler et al., 1978; Quick et al., 2002; Friedlander, 2007). These anatomical features include the location, size, and the supplying and drainage patterns of an AVM (Rangel-Castilla et al., 2015; Ozpar et al., 2019). In addition, DSA is also essential in endovascular procedures for treating AVMs by guiding the advancement of microcatheters and showing whether an endovascular therapy is completed or further interventions are required (Spetzler et al., 1987; Ozpar et al., 2019). The wealth of information in DSA, however, have not been used in *in silico* models for AVMs, which may cast light on some general hemodynamic characteristics of AVMs, such as the greatly increased flow across nidus, and the blood “steal” syndrome (Spetzler and Martin, 1986; Quick et al., 2002; Perez-Higueras et al., 2005; Friedlander, 2007; Weber et al., 2007). Still, the underlying mechanisms for AVM genesis, progression, and natural history are not well understood (Karlsson et al., 2018). For example, AVMs may rupture due to abnormal stress, but to which an extent would such a stress cause hemorrhage is still controversial (Gao et al., 1997; Rangel-Castilla et al., 2015).

A number of AVM models have been developed over the past two decades, in particular by Young and his co-workers (Gao et al., 1997; Massoud et al., 2000; Quick et al., 2002). Among these models, Gao et al. (1997) developed a theoretical framework that includes major cerebral vessels, based on which they simulated the blood flow in AVMs of different sizes, and also shear stress-induced dilation in conductance vessels. This model, however, is unable to explain the clinical observation that there is no correlation between the supplying arterial pressure and draining venous pressure (Young et al., 1994). Quick et al. (2002) improved the model of Gao et al. (1997) and further simulated the hypotension in the vicinity of AVM using an extranidal adaptation theory. In the models of Gao et al. (1997) and Quick et al. (2002), only a singular feeding artery and draining vein for an AVM were considered. In contrast, Hademenos et al. (1996) created a mathematical model to simulate an AVM with 4 feeding arteries, 2 draining veins, and 28 inter-connected plexiform and fistulous components. This model has the most complex intranidal structure among published models, to our knowledge. However, the cerebral vascular organization in Hademenos et al. (1996) does not strictly follow the cerebral anatomy. For example, the Circle of Willis (CoW), which is critical in distributing cerebral blood flow (CBF), was not incorporated into the model. In another study, Guglielmi used a simple electrical analog where the AVM nidus is composed of several parallel branches, and supplied by two feeding arteries and drained by one single vein (Guglielmi, 2008).

Great insights have been gained from the above *in silico* simulations, which are all lumped parameter models where the mass of vessels within an AVM are lumped into one or several electronic components to simulate the blood pressure gradient and flow through it (Hademenos et al., 1996; Guglielmi, 2008). However, it is often necessary to take into account the actual patient-specific anatomical features of an AVM, rather than idealistic topological designs. The aims of the study are to connect the analysis of DSA images with an *in silico* model for AVM and to build a platform where future AVM models can be based upon.

## Computational Modeling for AVM

### A Topologically Accurate Electrical Analog Model for Cerebral Circulation

#### Model Topology

**FIGURE 1:**
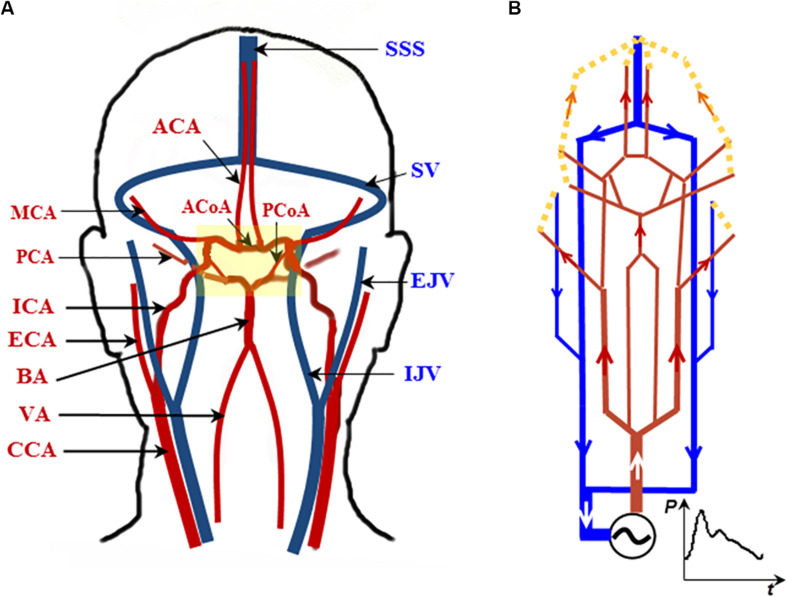
A topologically accurate cerebral circulation model. **(A)** The vessels in red are arteries; those in blue are veins. The CoW is highlighted in the yellow block (more details in Figure 2). **(B)** Schematic circuit of the cerebral circulation diagram in **(A)**. Abbreviations: CCA, ICA, and ECA—common, internal, and external carotid artery, respectively; BA—basilar artery; VA—vertebral artery; MCA, PCA, and ACA—middle, posterior, and anterior cerebral artery, respectively; SSS—superior sagittal sinus; SV—sigmoid vein; EJV and IJV—external and internal jugular vein, respectively. The generic model is based on Chau and Ho (2020).

We adopt a recently developed electrical analog model for the cerebral circulation model (Chau and Ho, 2020), whereby the topology of the cerebral vasculature is similar to that described in Gao et al. (1997) and Quick et al. (2002), but with additional external carotid artery (ECA) components included (Figure 1A). The model was implemented in Matlab/Simulink (Mathworks, Natick, MA, United States), with the layout shown in Figure 1B. Forty-nine major cerebral arteries and veins ranging from the aorta to the efferent arteries of the CoW and from the superior sagittal sinus (SSS) and the sigmoid veins (SV) to the jugular veins (JV) are included in the analog model.

**FIGURE 2:**
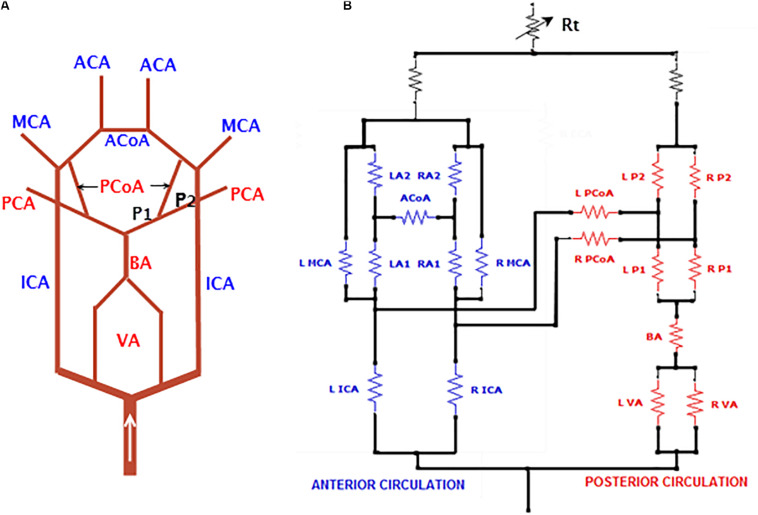
A subset of the electrical analog detailing the CoW. **(A)** The ACoA connects the left and right anterior circulation while the PCoA connects the anterior and posterior circulation. The PCoA splits PCA into P1 and P2 segments, and **(B)** implementation of the CoW in Simulink. For abbreviations of vessel names, refer to Figure 1.

A subset of this analog model, detailing the unique structure of CoW, is shown in Figure 2A. Note that the posterior communicating arteries (PCoAs) connect the anterior and posterior circulations: the vessels with names in blue are in the anterior circulation and those in red are in the posterior circulation (Figure 2). Specific to the AVM case to be described in this paper, PCoAs split the PCA into a P1 and a P2 segment. The cerebral flow in the CoW is very complex and prone to pathological conditions. Indeed, most of the arterial lesions, e.g., cerebral aneurysms and AVMs, occur in this region.

The resistance of a blood vessel is determined by Poiseuille’s law and is proportional to *L*/*r*^4^, where *L* and *r* are the length and radius of the vessel, respectively. The diameter and radius of blood vessels are adopted from literatures (Avolio, 1980; Hillen et al., 1986; Quick et al., 2002; DeVault et al., 2008). To convert the resistance and pressure into electronic units in the circuit, an empirical gain coefficient was used to relate the resistance with ohms, and another coefficient relates pressure with voltage in volts (Ho et al., 2013). The boundary condition for the model was a pulsatile aortic pressure adopted from Olufsen et al. (2000), which was digitized in its Fourier series of the first 10 frequencies.

This electrical analog model gives rise to a *generic* baseline model for cerebral circulation, where a complete CoW is assumed. The electrical circuit consists of 49 electronic components, among which 44 and 5 resistors represent cerebral arteries and veins, respectively. The circuit diagram is quite large and therefore is not displayed here but provided in the Supplementary file. In addition, the SimuLink source code for the generic model with all parameters is provided for the interested reader’s reference.

#### Autoregulation Model

An autoregulation mechanism is implemented for the vascular bed through a coupling of non-linear pressure-dependent resistor and an inductor. The inductor is used to damp the highly pulsatile arterial flow, whereas the non-linear resistor is used to regulate the CBF under acute hypertensive or hypotensive conditions (Chau and Ho, 2020). The governing equation for the non-linear resistor is:

(1) $$R_{t}=\left(1+\frac{P_{t}-P_{n}}{P_{n}}\right)\,R_{n}$$

where *R*_*t*_ is the transient resistance, is the pulsatile arterial pressure, *P*_*n*_ is the normal mean blood pressure (100 mmHg), and *R*_*n*_ is the resistance under a normal pressure range (80–120 mmHg).

**FIGURE 3:**
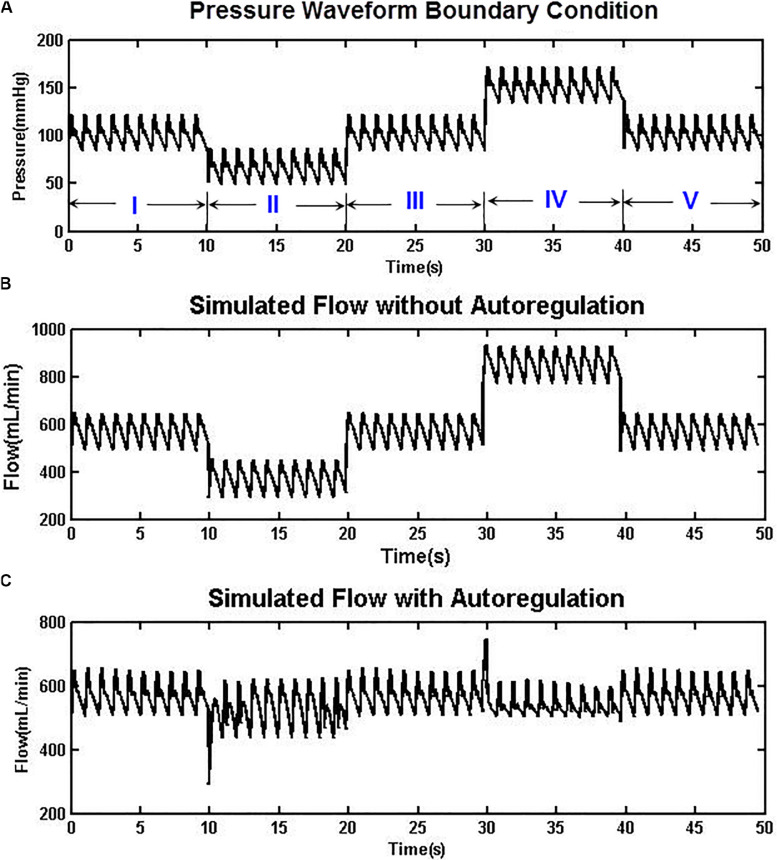
**(A)** An artificially designed aortic pressure waveform with normo-, hypo-, and hypertensive phases. **(B)** The simulated CBF without autoregulation varies between 350 and 850 ml/min. **(C)** With the autoregulation model implemented, the CBF was relatively stable at approximately 590 ml/min in both hypotensive and hypertensive phases.

The autoregulation mechanism of Eq. (1) was tested using an artificial pressure waveform, which contained five phases with 10 cardiac cycles for each phase (Figure 3A). A normal blood pressure range of 80–120 mmHg (100 mmHg mean) was arranged at the first, third, and fifth phases, whereas hypotensive and hypertensive conditions of 50–90 and 130–170 mmHg were placed at the second and fourth phases, respectively. The whole blood pressure range of 50–170 mmHg represents physiological pressure deviations that may be effectively autoregulated to maintain a steady CBF. Figure 3B shows the simulated cerebral flow rate waveforms without imposing the autoregulation mechanism. It can be seen that the mean flow rate varies between 350 and 850 ml/min, yet with the autoregulation mechanism introduced, as shown in Figure 3C, the CBF is able to recover from an initial oscillation and then maintain a relatively stable rate at approximately 590 ml/min (Chau and Ho, 2020).

### A Patient-Specific AVM Case Study

#### DSA Imaging

The Endovascular Intervention Unit of the Neurosurgery Department of the West China Hospital (WCH) treats approximately 70 AVM patients annually. Of these patients, about 50% receive surgeries following endovascular treatments. We randomly selected an AVM case for flow simulations.

**FIGURE 4:**
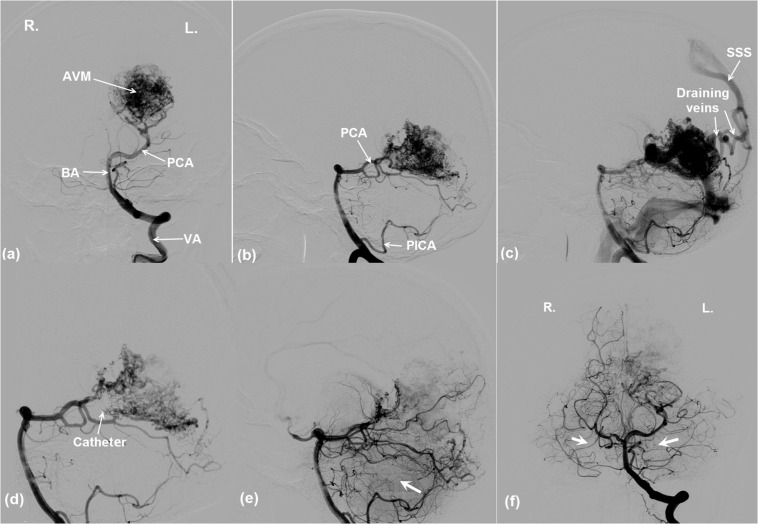
DSA images showing the angio-architecture of an AVM. **(a,b)** The left PCA and PICA are the feeding arteries of the AVM. **(c)** The AVM is drained by two veins that flow into SSS. Endovascular embolization of the AVM. **(d)** The thin arrow indicates the end of a microcatheter, where an embolization agent was being released. **(e,f)** The arrows indicate that ipsilateral and contralateral brain tissue perfusions were improved after AVM occlusion. Note the poor perfusion of the right and left brain pre-AVM embolization in **(a)** and **(d)**. Better perfusions are evident post-AVM embolization in **(e)** and **(f)**. For vessel abbreviations, refer to Figure 1.

An 18-year-old female patient was referred to the WCH with symptoms of eyesight loss of her right eye and headache. CT scans revealed left cortex deformation and a suspected AVM growth at the left occipital lobe. The AVM was confirmed by a DSA procedure (INTEGRIS Allura Flat Detector, Philips Medical Systems) (Figure 4). The left PCA and posterior inferior cerebellar artery (PICA) were identified as the feeding arteries of the AVM (Figures 4a,b). The two draining veins converge into the superior sagittal sinus (SSS) (Figure 4c). The vision loss of the patient’s right eye can be explained from the cerebral circulatory steal syndrome, where the AVM shunts the blood flow away from the left occipital lobe—the visual processing center for the right eye.

An endovascular embolism procedure was performed for the patient (Figure 4d). Intra-procedural DSA images in Figure 4d indicate that an embolization agent (Onyx 18, ev3, Irvine, California) was being released into the left PCA via a microcatheter. Figures 4e,f demonstrate that the AVM was partially occluded from the cerebral circulation, which immediately led to a better perfusion in the ipsilateral (Figure 4e) as well as the contralateral (Figure 4f) posterior brain regions. The phenomena are evident from the darker brain tissues shown in the image due to the presence of contrast agent.

### Adaptation of the Analog Model to the AVM Case

**FIGURE 5:**
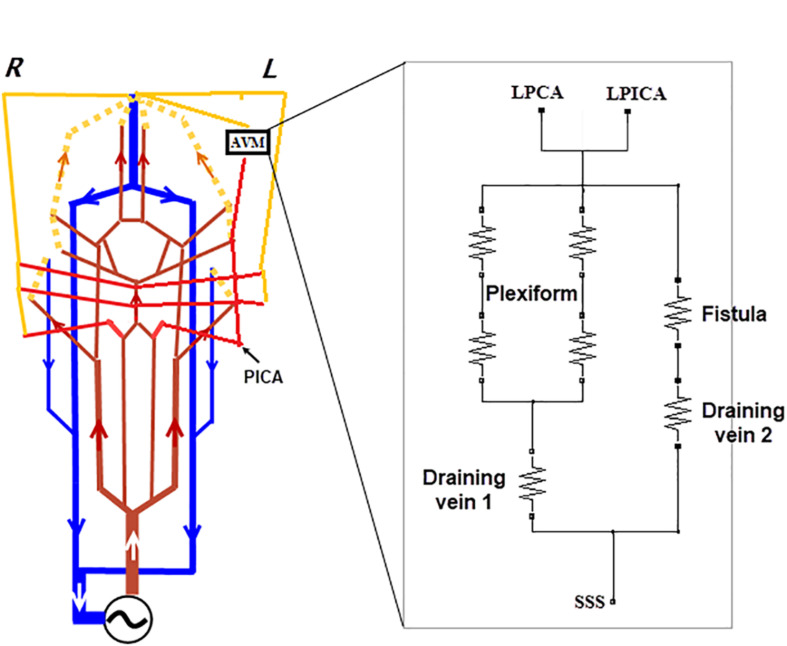
Modifications made to the generic circuit model of Figure 1. **(A)** Branches of the BA, i.e., SCA, AICA, and PICA were added to the circuit, and the left PICA connected to the AVM as one of the two feeding arteries. **(B)** An analog model for the AVM: a plexiform runs parallel with the arterial venous fistula. Two veins, per the DSA image in Figure 3c, drain the AVM.

The supplying and draining vessels revealed from DSA images were critical for the diagnosis and treatment of the AVM. The small vessels and the AVM nidus can be lumped into several electronic elements. In our implementation, the AVM was modeled by two parallel resistors with equal resistance to simulate the AVM plexiform. Another parallel branch consisting of a resistor with much lower resistance was used to mimic the fistula (Figure 5). This arrangement is similar to that described in Guglielmi (2008) but with fewer components.

Since the feeding arteries arise from the left PCA and PICA, the left PCA is rerouted onto the nidus network, bypassing the posterior capillary bed, and adjoining to the AVM nidus (see the DSA image in Figure 4b). The PICA was the other supplying artery of the AVM from the vertebral artery and was implemented in the analog model. In addition, the anterior inferior cerebellar artery (AICA) and the superior cerebellar artery (SCA) were added into the circuit following the vascular anatomy. The above modifications are illustrated in Figure 5. The outlet of the AVM is connected to two draining veins of equal resistance, which ultimately converge into the SSS. This topological arrangement matches that revealed in the DSA images (see Figure 4c).

In the current implementation, the AVM does not have an intranidal adaptation mechanism, following what is suggested in literature (Quick et al., 2002). In other words, the AVM does not possess regulation functions *per se*. On the other hand, extranidal regulation is simulated and included in the global autoregulation function in the same manner as the normal case, i.e., regulated by Eq. (1).

## Results

### The Baseline Generic Model

**FIGURE 6:**
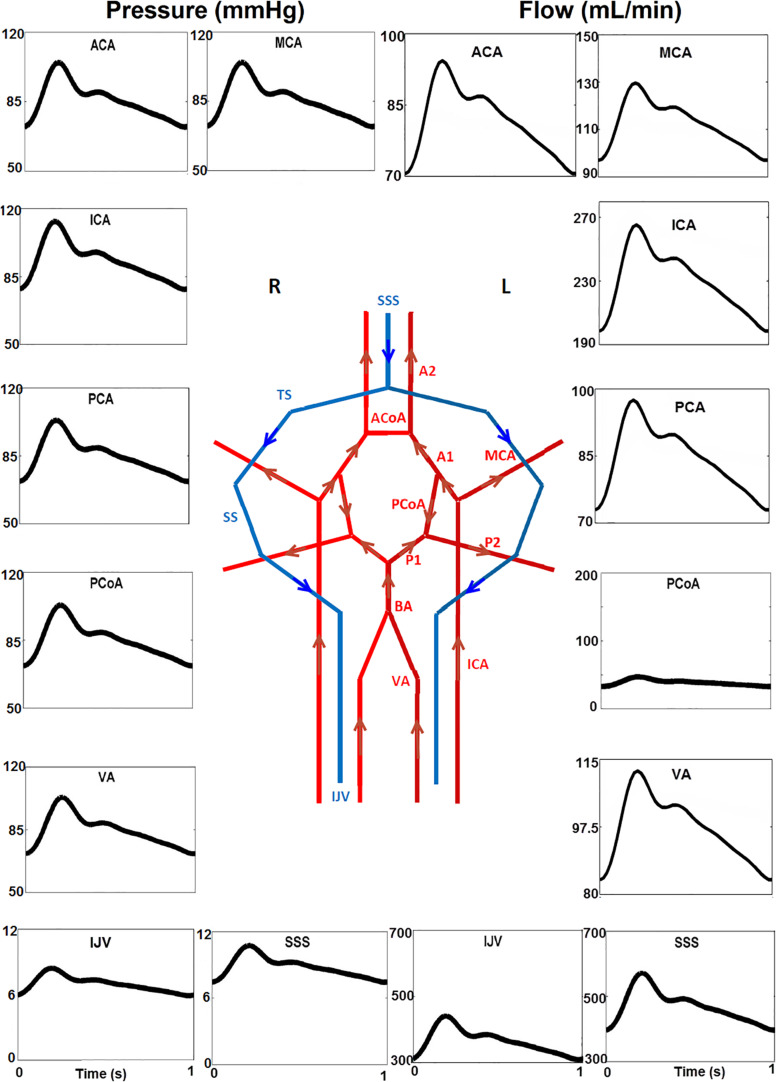
Temporal blood pressure and flow rate profiles at key sites in the arterial (ACA, PCA, ACoA, and PCoA) and venous (SSS, SS, and IJV) networks. Note that the flow direction of PCoA was from the anterior to posterior regions in the generic model, and that the venous pressure (6–11 mmHg) is much lower than that in arteries. Furthermore, the scales for the *Y*-axis are different.

The baseline model shown in Figures 1, 2 was solved over 50 cardiac cycles. The profiles of flow rate and blood pressure at several typical sites of arteries and veins are shown in Figure 6. Blood flows in large cerebral arteries (ACA, MCA, and PCA) are pulsatile and they have a similar pressure waveform profile (75–115 mmHg). The blood pressure is much lower and steady in veins (6–11 mmHg). The total CBF (the sum of flow rates through the left and right ICAs and VAs) was ∼660 ml/min, close to the 700 ml/min theoretical CBF, i.e., 15% of cardiac output of ∼5 L/min. In particular, the flow rate through the anterior circulation was 460 ml/min, i.e., ∼70% of cerebral flow, consistent with clinical reports that the anterior circulation contributes to ∼75% of CBF, and VAs provide the remaining ∼25% of CBF (Zagzoule and Marc-Vergnes, 1986; Gao et al., 1998). There is a small flow rate (∼40 ml/min) in the PCoA, indicating the blood flow and distribution between the anterior and posterior brain regions. This feature of blood flow distribution in the CoW may change due to vascular pathology in the cerebral arteries or incomplete CoW (David et al., 2003).

### Flow Simulations for the AVM

**FIGURE 7:**
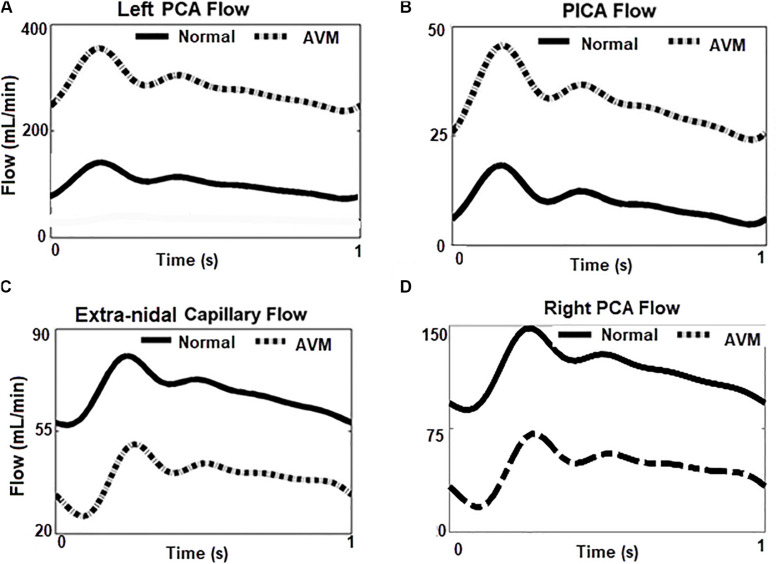
Flow rates at different locations in the circuit with/without AVM. **(A,B)** Blood flows increase greatly in the left PCA and PICA, the feeding arteries of AVM. **(C,D)** Blood flow reduced in ipsilateral capillary bed, and the contralateral right PCA.

With the introduction of a left AVM in the model, the flow rates at the left PCA, PICA, and the right PCA are shown in Figure 7. The shunting effects of the AVM are obvious: the flow rate in the left PCA is much higher than the normal case without AVM (300 ml/min vs 100 ml/min) (Figure 7A), because the left PCA now serves as a supplying conduit to the AVM. Since extranidal tissues have a capillary bed where the size of capillaries (diameter ∼10 μm) is much smaller than the vessel inside AVM nidus (∼100 μm), the resistance to blood flow is much higher in extranidal tissues according to Poiseuille’s law. As a result, the blood perfusion to adjacent extranidal brain tissues is drawn to this conduit to AVM. Indeed, the high flow rate (300 ml/min) through AVM accounts for nearly half (43%) of the total intracranial flow (∼700 ml/min). In comparison, Nornes and Grip measured 150 to 900 ml/min (average 490 ml/min) from nine AVM patients (Nornes and Grip, 1980). Consequently, the blood flow into the extranidal posterior capillary network was drastically decreased from 70 to 30 ml/min.

Compared to the left PCA (diameter ∼2 mm), the diameter of the left PICA (diameter ∼1 mm) is smaller, and its contribution to the AVM flow is not as significant as the PCA. However, the flow rate (∼35 ml/min) in the left PICA is much higher than the normal value of 10 ml/min (Figure 7B), leading to a higher shear stress along the vessel. This may pose a higher hemorrhage risk than that induced by a normal shear stress (Meng et al., 2014).

A question raised is whether the circulation “steal” effects are located in the vicinity of the AVM (Figure 7C) or they also influence other brain regions. From Figure 7D, it can be seen that the flow rate in the right PCA, i.e., the contralateral side of the left PCA, is much lower (30 ml/min vs 100 ml/min) when the AVM was included in the circuit. An inspection of the DSA images in Figures 4a,f reveals the phenomenon that the perfusion of the right hemisphere before the AVM embolization procedure was weaker than that after the procedure, as evident from the darker tissue signal after procedure (Figure 4a). Thus, the “blood steal” effects induced by the AVM at the left brain also affect the right brain.

**FIGURE 8:**
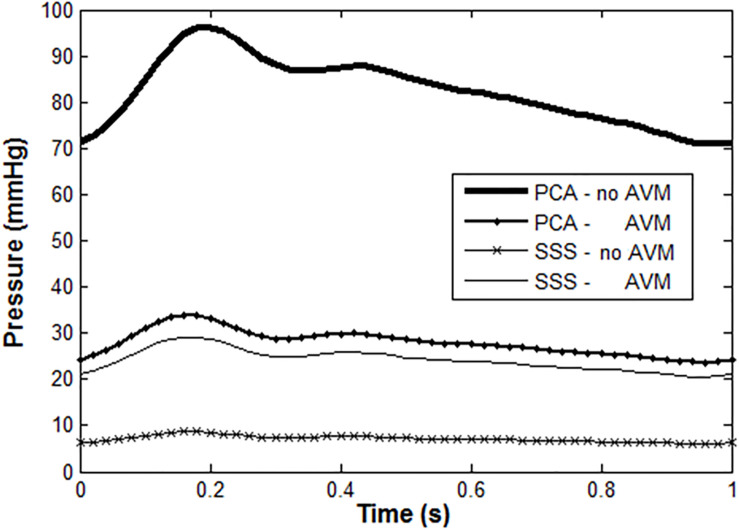
Blood pressure in PCA and SSS with/without AVM: normal PCA pressure is high and pulsatile under healthy condition, but much lower with the presence of AVM. In contrast, the venous pressure is much higher than normal when there is an AVM.

We also compared the blood pressures proximal and distal to the AVM, i.e., in the feeding artery, the left PCA, and the draining vein SSS. The results are shown in Figure 8. Without the presence of the AVM, the blood pressure in the PCA and the draining SSS was about 85 and 9 mmHg, respectively. With the introduction of AVM in the model, the blood pressure at the PCA is reduced to about 30 mmHg, whereas the pressure at the SSS is raised to about 22 mmHg. The simulation results are consistent with many clinical observations, e.g., in Spetzler et al. (1987); Kader et al. (1994), and Nornes and Grip (1980) where the presence of AVM “drags” down the arterial pressure at its proximal entrance, but “pulls” up the venous pressure at its distal outlet. Specifically, Kader et al. reported the draining venous pressure as 21 ± 10 mmHg, and there were no substantial differences in hemorrhage and non-hemorrhage groups (Kader et al., 1994).

## Discussion

Advanced imaging technologies nowadays have revealed more AVM cases, including many that are asymptomatic (Friedlander, 2007; Rangel-Castilla et al., 2015). Clinical decisions need to be made concerning whether to treat an AVM invasively or leave them intact after weighing the surgical risks and benefits (Weber et al., 2007; Rangel-Castilla et al., 2015). In the surgical planning process, cerebral hemodynamics pre- and post-AVM resection is one of the core factors to be considered, and it is still not well understood (Rangel-Castilla et al., 2015). For example, there are both favoring and contradicting clinical observations against the “normal pressure perfusion breakthrough” (NPPB) hypothesis proposed by Spetzler et al. (1978) in 1978, which states that the tissues adjacent to AVM have a higher risk of hemorrhage after AVM resection, due to loss of regulation functions under chronic hypotension (Rangel-Castilla et al., 2015).

It has been shown that *in silico* models can provide useful insights in certain aspects of AVM, such as the extranidal adaption to hypotension (Quick et al., 2002) and the NPPB theory. The former mechanism suggests that extranidal brain tissues may still receive sufficient blood flow despite being under chronic hypotensive conditions (Quick et al., 2002). The latter mechanism interprets the phenomena of edema and hemorrhage that may occur in brain tissues adjacent to AVM after a surgical resection, owing to loss of autoregulation (Spetzler et al., 1978; Rangel-Castilla et al., 2015).

However, it is still controversial whether regional regulation functions may be deprived due to the presence of an AVM, or just their lower limits are displaced (Quick et al., 2002). An updated theory about the edema and hemorrhage phenomena after AVM resection is the so-called “occlusive hyperemia theory” proposed by Al-Rodhan et al. in 1993 (Al-Rodhan et al., 1993). This theory is based on venous occlusion and stasis of flow in feeding arteries of previously resected AVMs (Rangel-Castilla et al., 2015).

Our model provides certain support to the hyperemia theory. We have shown in the transnidal pressure simulation (Figure 7) that there is a much higher pressure and flow rate at the draining veins before AVM resection. In the cardiovascular system, blood vessels remodel themselves particularly in the medial layer to provide the mechanical strength and tone for increased flow stress and pressure (Tarbell et al., 2014). After AVM resection, however, the remodeled draining vessel constricts due to a lower pressure and flow rate in it and the high vascular tone, consequently leading to venous occlusion. Blood flow therefore is diverted to the previous extranidal tissues, potentially causing edema and hemorrhage. The fact that AVM rupture accounts for 2% of all strokes necessitates more basis and clinical research in the pathophysiology of AVM (Friedlander, 2007).

The novelty of the work is that it is the first AVM model that incorporates subject-specific information from DSA image. Since the morphological arrangement of the AVM in the cerebral circulation model is subject-specific, a different AVM patient requires having an AVM model customized and re-adapted in the generic 0D model to account for the specificity.

There are some limitations pertaining to the current model. Firstly, the implementation of the autoregulation mechanism is rather simple. The model assumes that the CBF responds to blood pressure variations only, yet CBF autoregulation is a complex process that involves neurogenic, metabolic, and myogenic mechanisms (Rangel-Castilla et al., 2015). Secondly, the electrical circuit itself represents a highly simplified analog model that uses resistors for blood vessels, neglecting the non-linear elasticity of the wall. Having capacitors and inductors included for the large arteries may improve the simulations, but will inevitably increase the complexity of the model. Another development direction is to apply a coupled or hybrid electrical analog and one dimensional (0D–1D) model, which has been presented in recent multidimensional modeling works, e.g., in Baker et al. (2020) and Yu et al. (2018). Specifically, the CBF re-distribution in the CoW could be analyzed from a 1D flow model coupled with 0D models at vessel outlets (Yu et al., 2018). Prediction of blood flow through the AVM is dependent strongly on the parameter values involved in the model. While we partly proved the rationality of the model by comparing model predictions with clinical data, the simulations may not fully represent the hemodynamic features for the patient investigated in the study due to lack of *in vivo* data. This is another important limitation of the current study.

It is also worth noting that while our model results were compared to DSA data, the calibration was rather phenomenological than quantitative. In *in vivo* measurements such as CBF velocities measured using transcranial ultrasound (Diehl et al., 1994) or phase-contrast MRI, blood pressure measured invasively with catheter or non-invasively with blood pressure device would be ideal to validate the simulation results but unfortunately were not available in this study. The clinical practices in the WCH for suspected symptomatic AVM cases usually start with a CT scan for diagnosis, followed by a DSA detection if confirmation is required. DSA is also performed during an endovascular procedure. However, intra-procedural, vessel-specific flow data are not measured other than vital bio-signals. In-depth investigations of patient-specific AVMs would require a careful design of the clinical workflow for the data to validate models, and with an ethic approval. In addition, while the diameters and lengths of major cerebral arteries could be extracted from CT angiography or MR angiography images, these images were not available for 3D reconstruction for the patient. Hence, vessel diameters and lengths were adopted from literatures, e.g., from Hillen et al. (1986).

Nevertheless, the presented model provides an *in silico* platform for further AVM investigations. An immediate future work would be to numerically investigate the embolization of AVMs and those AVM cases located at different regions of the brain, e.g., in the anterior circulation region. Indeed, AVM occurs at the anterior circulation in 70–80% of the time (Friedlander, 2007). Another future direction will require incorporating regional regulation mechanisms into the model. The ultimate aim is to better understand and to better predict rupture risks of AVM, and thus aid clinical decision making.

## Conclusion

In this study, we used the information from DSA images of an AVM patient to alter an electrical analog model for cerebral circulation. Our simulations reproduced multiple flow phenomena that can be observed from the DSA images. We suggest the current *in silico* model be used as the basis for further AVM investigations.

## Data Availability Statement

All datasets generated for this study are included in the article/Supplementary Material.

## Ethics Statement

The studies involving human participants were reviewed and approved by The Ethics Committee of the West China Hospital, Sichuan University. The patients/participants provided their written informed consent to participate in this study.

## Author Contributions

CZ performed the endovascular procedure of the AVM case and provided clinical insight. NC made the numerical simulation. HH drafted the manuscript. All authors reviewed and agreed the current form of the manuscript.

## Conflict of Interest

The authors declare that the research was conducted in the absence of any commercial or financial relationships that could be construed as a potential conflict of interest.
