# Supplementary material for: Patient-Specific Blood Flow Analysis for Cerebral Arteriovenous Malformation Based on Digital Subtraction Angiography Images
Source: Front Bioeng Biotechnol. 2020 Jul 23;8:775. doi: 10.3389/fbioe.2020.00775 (PMC7390970; doi:10.3389/fbioe.2020.00775)
Supplement: Supplementary file 1 [file Data_Sheet_1.docx]

**Supplementary Document**

**“Patient-specific Blood Flow Analysis for Cerebral Arteriovenous Malformation Based on Digital Subtraction Angiography Images”**

**By C. Zhang, C. Chau, and H. Ho**

**A.1. Cerebral Vascular Anatomy**

Cerebral blood flow (CBF) is supplied to the brain via a pair of carotid arteries and a pair of vertebral arteries (refer to Fig. 1 of the paper). The two common carotid arteries (CCA) bifurcate into the internal and external carotid arteries (ICAs and ECAs). The ICAs branch into anterior cerebral arteries (ACAs) and middle cerebral arteries (MCAs) to supply the anterior circulation. The ECAs develop extracranially and deliver blood to the face and scalp. The two vertebral arteries (VA) merge into the basilar artery (BA), which splits into the left and right posterior cerebral arteries (PCAs) to supply the posterior circulation. These arteries may be connected by communicating arteries, i.e., the anterior communicating artery (ACoA) to connect the left and right hemispheres, and the posterior communicating artery (PCoA) to connect the anterior and posterior circulations (ACAs/MCAs and PCAs), and thus complete the CoW.

Large cerebral veins include the superior sagittal vein (SSS), which distributes venous flow into the left and right sigmoidal veins (SV). The SVs are connected to a pair of internal jugular veins (IJVs), which confluence with the external jugular veins (EJVs) and other veins into the superior vena cava (SVC), and eventually return the blood back to the heart (see Fig. 1 of the paper).

**A.2. Electrical analog model**

We used the software Simulink, specifically the SimPowerSystems expansion, of Matlab (Mathworks, Natick, MA, USA) to implement the electric circuit of Fig. 1(b), and to perform simulations. We refer interested readers to the SimuLink source code attached as a supplementary file. In the circuit, various hemodynamic data are monitored by voltage and current meters in the circuit.

The arrangements of the electronic components were made in accordance with the actual anatomy. For example, as shown in Fig. 1(b), the ACoA connects the left and right ACAs, and separates the ACAs into A1 and A2 segments. Likewise a PCA separates the PCA into P1 and P2 segments. The terminologies for the vessels refer to that being used in the clinical context (e.g., in neuro-surgeries and endovascular interventions). For example, the basilar artery and its branches are shown in Fig. A1. External carotid arteries (ECAs) were separated into four segments, i.e., the lingual artery, the occipital and facial artery, the auricular artery, the maxillary and temporal arteries.

A subset of this circuit, detailing the CoW, is shown in Fig. 2 in the manuscript. Note, that the anterior and posterior circulations, which are sourced from the carotid and vertebral arteries respectively, are connected by the left and right posterior communicating arteries (PCoAs). PCoAs split the PCA into P1 and P2 segments. Fig. 2(b) shows the corresponding CoW circuit implemented in SimuLink. The nonlinear resistor *Rt*, governed by Eq. (1), is used to autoregulate the cerebral flow.

The source code of Simulink of the electrical analog model (in mdl format) with all parameters are provided as a separate supplementary file. This circuit gives rise to a *generic* baseline model for cerebral circulation, where a healthy, complete cerebral vasculature is assumed. The actual implementation of the circuit consists of 49 electronic components, among which 22 resistors represent intracranial arteries, 22 resistors represent extra-cranial arteries, 5 resistors represent veins, and a resistor/inductor couple to represent the vascular bed.

**A.3. Basilar and its branches in the modified circuit**

Alterations to the analog circuit can be made in several ways to simulate different AVM scenarios. Firstly, the circuit topology can be modified. Secondly, the parameters for electronic components can be changed. In the following section we use a patient case study to exemplify this process.


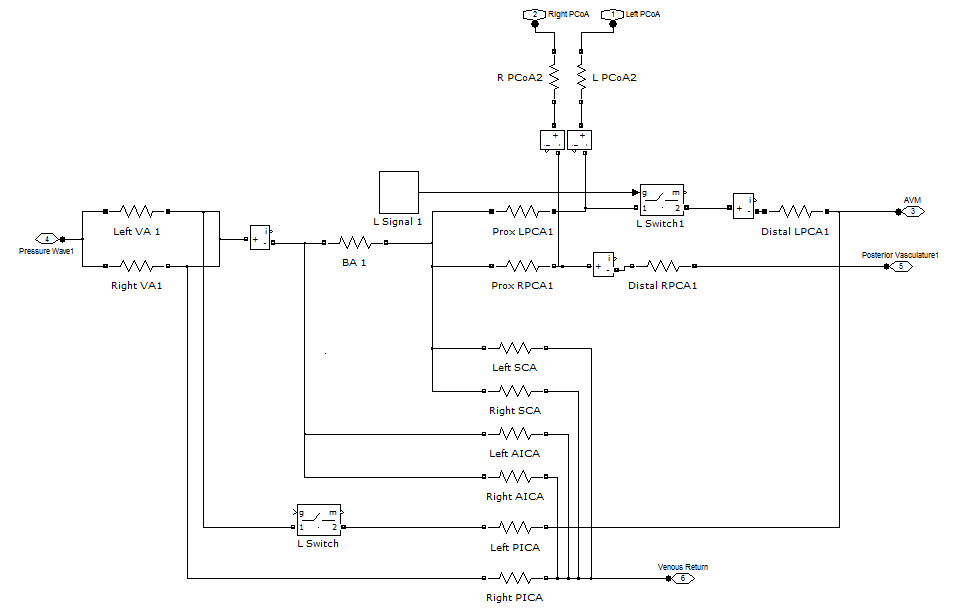


Fig.A1: Illustration of the basilar artery and its branches: the left PICA was connected to AVM. Note that switches are used to simulate feed artery embolism effects.

**A.4. Electronic component parameters**

In principle, the value of a single resistor was determined by the Poiseuille’s Law:

$R=\frac{\Delta P}{Q}=\frac{8\eta L}{\pi r^{4}}$ (1)

where R, Q, ΔP, r, L are the resistance, flow rate, pressure gradient, radius and length of the vessel, respectively. η is the dynamic viscosity of the blood. However, because the Poiseuille’s Flow law is not directly analogous to electrical components, an empirical gain coefficient had to be set to correlate the resistance with Ohms. Here the simulation data of interest were voltage and current which were analogues to blood pressure and flow, respectively. Their values are monitored by pressure/flow meters arranged in the circuit.

Typical diameters and lengths of these blood vessels in healthy adults have been reported in literatures, e.g., in references contained herein. These values can be used to gauge the resistance to blood flow. In this work they are used as in Table A1.

Table A1: Nominal resistance of blood vessels in the model. Vessel parameters calculated from cerebral vascular data in (Hillen et al., 1986) (Avolio, 1980) (DeVault et al., 2008) (Gao et al., 1997) (Quick et al., 2002) (Ho et al., 2013)

| **Name of vessel** | **Resistance (Ohms)** |
| --- | --- |
| **Arteries** |  |
| CCA | 425 |
| ICA | 1000 |
| VA | 500 |
| BA | 500 |
| MCA | 1150 |
| ACoA | 2000 |
| PCoA | 1750 |
| ACA (A1) | 500 |
| ACA (A2) | 400 |
| PCA (P1) | 100 |
| PCA (P2) | 575 |
| ECA Seg 1 | 2500 |
| ECA Seg 2 | 1000 |
| ECA Seg 3 | 1000 |
| ECA Seg 4 | 1000 |
| Temporal | 1000 |
| Maxillary | 1000 |
| Facial | 1000 |
| Occipital | 1000 |
| Lingual | 2000 |
| Auricular | 1000 |
|  |  |
| **Veins** |  |
| IJV | 10 |
| EJV | 2500 |
| SSS | 50 |
| Sigmoid sinus | 50 |

Note, that vessel diameter and length are subject-specific, and their values vary with different literatures. Therefore the data shown in this Table A1 should be treated as reference only.

**Reference**

Avolio, A. P. (1980). Multi-branched model of the human arterial system. *Med. Biol. Eng. Comput.* 18, 709–718. doi:10.1007/BF02441895.

DeVault, K., Gremaud, P. A., Novak, V., Olufsen, M. S., Vernières, G., and Zhao, P. (2008). Blood Flow in the Circle of Willis: Modeling and Calibration. *Multiscale Modeling & Simulation* 7, 888–909. doi:10.1137/07070231X.

Gao, E., Young, W. L., Ornstein, E., Pile-Spellman, J., and Qiyuan, M. (1997). A Theoretical Model of Cerebral Hemodynamics: Application to the Study of Arteriovenous Malformations. *Journal of Cerebral Blood Flow & Metabolism* 17, 905–918. doi:10.1097/00004647-199708000-00009.

Hillen, B., Hoogstraten, H. W., and Post, L. (1986). A mathematical model of the flow in the circle of Willis. *Journal of Biomechanics* 19, 187–194. doi:10.1016/0021-9290(86)90151-X.

Ho, H., Mithraratne, K., and Hunter, P. (2013). Numerical Simulation of Blood Flow in an Anatomically-Accurate Cerebral Venous Tree. *IEEE Transactions on Medical Imaging* 32, 85–91. doi:10.1109/TMI.2012.2215963.

Quick, C. M., Leonard, E. F., and Young, W. L. (2002). Adaptation of Cerebral Circulation to Brain Arteriovenous Malformations Increases Feeding Artery Pressure and Decreases Regional Hypotension. *Neurosurgery* 50, 167–175. Available at: http://journals.lww.com/neurosurgery/Fulltext/2002/01000/Adaptation_of_Cerebral_Circulation_to_Brain.25.aspx [Accessed September 9, 2013].
